# Novel Surgical Reconstruction Using a 3D Printed Cement Mold Following Resection of a Rare Case of Proximal Ulna Osteosarcoma: A Case Report and Description of the Surgical Technique

**DOI:** 10.3390/curroncol32080411

**Published:** 2025-07-22

**Authors:** Abdulrahman Alaseem, Hisham A. Alsanawi, Waleed Albishi, Ibrahim Alshaygy, Sara Alhomaidhi, Mohammad K. Almashouq, Abdulaziz M. AlSudairi, Yazeed A. Alsehibani, Abdulaziz O. Almuhanna

**Affiliations:** 1 Department of Orthopedic Surgery, College of Medicine, King Saud University, Riyadh 1211, Saudi Arabia; abalaseem@ksu.edu.sa (A.A.); halsanawi@ksu.edu.sa (H.A.A.); dralbishi@gmail.com (W.A.); ialshaygy@ksu.edu.sa (I.A.); mashouq2@gmail.com (M.K.A.); ab.alsudairi@hotmail.com (A.M.A.); yazeed.alsehibani@gmail.com (Y.A.A.); 2 College of Medicine, King Saud University, Riyadh 1211, Saudi Arabia; 3 Department of Orthopedics Surgery, King Saud Medical City, Riyadh 1211, Saudi Arabia; a.o.almuhanna@gmail.com

**Keywords:** osteosarcoma, proximal ulna, 3D printing

## Abstract

Osteosarcoma is one of the most common primary bone malignancies, typically occurring around the knee. However, the forearm is a rare site, with tumors in the proximal ulna being extremely uncommon. Primary sarcoma in this location presents a surgical challenge due to the complex anatomy and limited reconstructive options. We report a rare case of a 19-year-old female with non-metastatic, high-grade giant cell-rich osteosarcoma involving the right proximal ulna. To our knowledge, this is only the second reported adult case of this histological subtype in this location. The patient was treated at a specialized oncology center with neoadjuvant and adjuvant chemotherapy, along with wide intra-articular resection for local tumor control. Reconstruction was achieved using a novel, customized 3D-printed articulating cement spacer mold with plate osteosynthesis. Artificial elbow ligamentous reconstruction was performed using FiberTape and FiberWire sutures passed through drill holes, and the triceps tendon was reattached to the cement mold using an endobutton. This cost-effective and personalized surgical approach allowed successful joint reconstruction while maintaining elbow stability and function. Our case highlights a feasible reconstructive option for rare and anatomically challenging osteosarcoma presentations, contributing to the limited literature on proximal ulna giant cell-rich osteosarcoma.

## Simple Summary

Osteosarcoma is a serious type of bone cancer that usually affects the knee. When it occurs in less common locations like the ulna (one of the forearm bones), it becomes harder to treat due to the area’s complex anatomy and fewer established surgical options. In this report, we describe the case of a young adult patient with a rare form of osteosarcoma in the upper part of her ulna. Because traditional metal implants were not available and treatment could not be delayed, the surgical team used 3D printing technology to design a custom mold. This mold was used to shape a cement-based implant that matched the patient’s anatomy and allowed for functional reconstruction of her elbow. The patient recovered well and maintained good arm function over two years of follow-up. This case shows how 3D printing can offer affordable, personalized solutions in rare and urgent cancer surgeries, especially where resources are limited.

## 1. Introduction

Osteosarcoma is the most common malignant osseous tumor, with a reported global incidence of 3.4 million per year. Osteosarcoma mostly occurs in young adults within the age group of 10–25 years [1]. Giant cell-rich osteosarcoma (GCRO) is a rare subtype of high-grade osteosarcomas, accounting for merely 1–3% of all osteosarcomas. The diagnosis of GCROs can be elusive and challenging, as the radiological and histopathological features are quite similar to other benign and malignant giant cell tumors [2]. Conventional osteosarcoma most commonly affects the distal femur, proximal tibia, and proximal humerus, with wrist involvement being extremely rare [3]. In addition, the forearm is a rare site of occurrence for an osteosarcoma. Primary tumors of the ulna comprise <1% of all lesions and <2% of all bone lesions [1]. Due to the very unusual locations of these tumors, they pose significant reconstructive surgical challenges because of the complex anatomic relationship between the bony and surrounding capsuloligamentous structures, as well as the limited availability of optimal reconstructive options, which are vital for elbow stability and function. Hence, addressing the defects following surgical resection of these tumors is an exceptionally difficult task, as previously described in the literature [4].

We report a rare case of a 19-year-old female patient diagnosed at our hospital with a high-grade GCRO of the right proximal ulna without intra-articular involvement. The case describes a novel cost-effective reconstructive surgical option utilizing a customized 3D-printed cement spacer molded to match the patient’s own anatomy, alongside the conventional standard management protocol of osteosarcoma, which involves local control via wide surgical resection and systemic neoadjuvant as well as adjuvant chemotherapy for osteosarcoma. This case contributes to the limited literature on proximal ulna GCRO by presenting an innovative, cost-effective reconstruction technique that may serve as a feasible alternative in resource-limited settings.

## 2. Case Presentation

**Figure 1:**
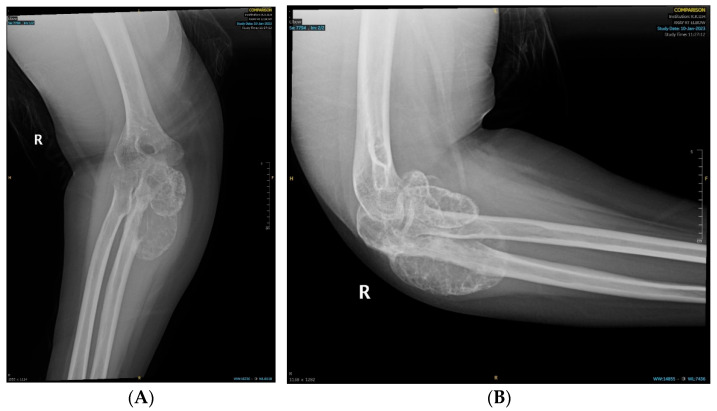
Radiographs depicting the ulnar bony lesion: (**A**) anteroposterior view; (**B**) lateral view.

We report the case of a 19-year-old female patient who was referred to the orthopedic oncology serviceat our hospital. She had been complaining of intermittent, mild right elbow pain for the past two years, which had progressively worsened. Subsequently, she started experiencing a decreased range of motion about six months prior to her presentation to our clinic, prompting her to seek medical attention. She developed a painful mass over her right elbow one month before her visit to our clinic. The swelling had been increasing in size, causing progressive pain and discomfort, and limiting her range of motion. She denied any history of trauma, sports injury, or infection in her elbow, and she had no constitutional symptoms. Her past medical, personal, or family history was unremarkable. On examination, there was mild tenderness over the olecranon and lateral epicondyle of the humerus, with no overlying skin changes. Her elbow flexion–extension range of motion was limited to 90° to 130° due to a mechanical block by the tumor and associated swelling; however, she had a full range of pronation and supination. Ulnar nerve function was intact, as were the median, radial, and musculoskeletal nerves, and distal pulses were palpable. No palpable regional or ipsilateral axillary lymphadenopathy was noted. A radiograph of the right elbow showed a poorly demarcated destructive bony lesion involving the proximal ulna with mixed lytic and blastic matrix and soft tissue extension but no pathologic fracture or involvement of adjacent radius or humerus (Figure 1). These findings were worrisome and highly suspicious for a primary bone malignancy, which warranted further evaluation with advanced imaging by magnetic resonance imaging with Gadolinium contrast (to evaluate the locoregional extent of the tumor), as well as an image-guided Tru-cut core needle biopsy for histopathologic diagnosis and systemic staging via computed tomography (CT) scans of the chest, abdomen, and pelvis, and a whole body nuclear study (to ensure no metastatic disease) in addition to laboratory investigations.

**Figure 2:**
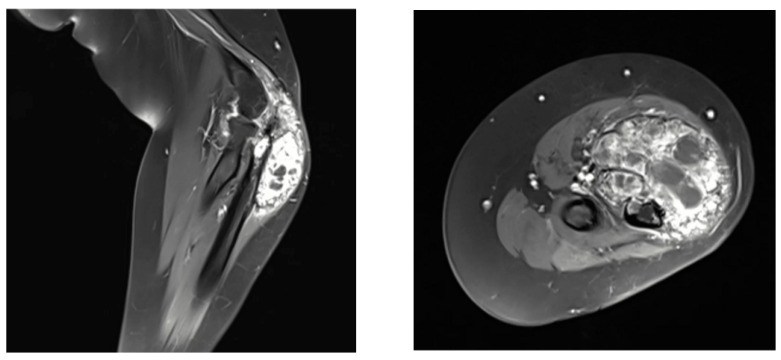
Magnetic resonance imaging (MRI) of the right forearm showing the giant cell-rich osteosarcoma (GCRO).

MRI of the right forearm revealed an aggressive, heterogeneous osteolytic lesion with post-contrast enhancement and surrounding soft tissue edematous changes with no evidence of intra-articular involvement or nearby adherence to neurovascular structures (Figure 2).

An image-guided Tru-cut core needle biopsy revealed a high-grade, giant cell-rich osteosarcoma.

Local and systemic staging evaluations confirmed a localized non-metastatic disease. Therefore, she was referred to the medical oncology service in our oncology center, and a multidisciplinary sarcoma tumor board meeting discussion recommended the standard management protocol for osteosarcoma, which includes neoadjuvant systemic chemotherapy (three cycles of doxorubicin and cisplatin), followed by restaging and local surgical control with wide resection and reconstruction, then subsequent adjuvant chemotherapy.

Restaging radiologic investigations following neoadjuvant chemotherapy were performed, including MRI of the right elbow, which did not show any significant interval post-therapy changes or progression of the tumor. In addition, CT scans of the chest, abdomen, and pelvis did not reveal any intrathoracic or abdominopelvic metastasis.

### 2.1. Surgical Technique

The surgical procedure involved three main stages: (1) wide intra-articular resection of the proximal ulna tumor for limb salvage, (2) reconstruction of the proximal ulna using a 3D-printed cement-molded articulating spacer augmented with plate osteosynthesis, and (3) elbow ligamentous reconstruction using FiberTape and FiberWire sutures. Preoperative planning of tumor resection length was based on the post-chemotherapy MRI scan. The measured length of the tumor was 9 cm plus an additional 3 cm as a safe margin.

**Figure 3:**
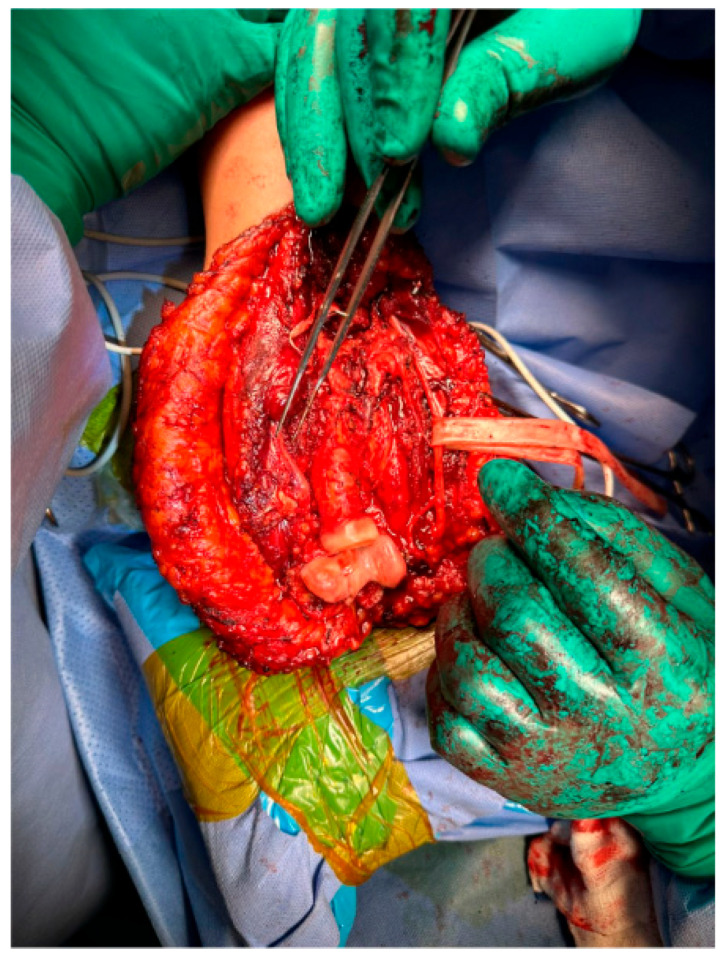
Wide resection of the right proximal ulna.

**Figure 4:**
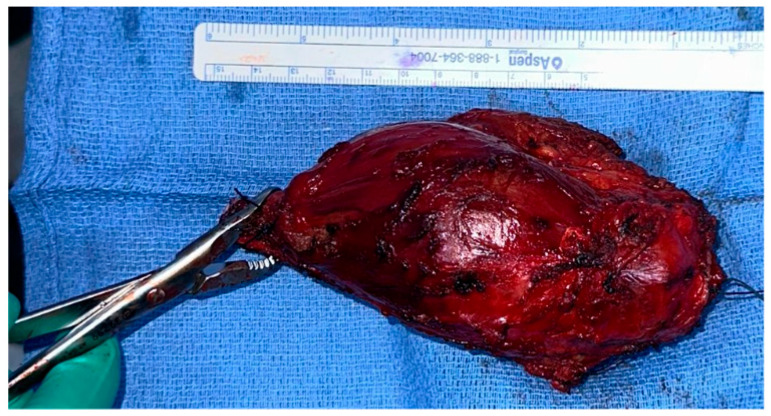
Complete excision of the tumor with the surrounding tissue margin.

The surgical procedure was performed under general anestesia, and a prophylactic 2 g intravenous cefazolin antibiotic was administered as per standard recommendations. The patient was put in a supine position with the right arm across the chest. Prepping and draping of the right upper extremity were performed, followed by repeat prepping after draping with chlorhexidine. Posterior skin incision was marked with an elliptical shape around the biopsy tract. Then, wide and thick medial and lateral subcutaneous flaps were elevated from the distal humerus to the distal ulna. The ulnar nerve was identified and protected throughout the procedure. The triceps and anconeus muscles were reflected off the ulna, leaving a cuff attached to the ulna for a safe margin (this was conducted medially to laterally, leaving the anconeus in continuity with the triceps). Next, the common flexor tendon origin was released, tagged, and reflected from the humerus for exposure. Then, the lateral and medial collateral ligament (LUCL, MCL) and capsule were released from the humerus side. Subsequently, osteotomy was carried out at the proximal ulnar diaphysis as planned preoperatively, at 12 cm from the tip of the olecranon and 9 cm from the radioulnar joint, after circumferentially releasing the soft tissue at the osteotomy site. Next, a bone marrow biopsy was obtained from the distal side of the ulnar stump and sent as a frozen section for histopathology assessment, which confirmed a negative bone marrow margin. We then turned our attention to the radial side of the proximal ulna by releasing the interosseous membrane and muscles, leaving a cuff for a safe margin. We completed the dissection along medial attachments up to the proximal radioulnar joint level, as well as the elbow joint capsule, which was circumferentially released from the distal humerus and radial head sides until complete resection of the proximal ulna tumor was achieved, and the specimen was sent for histopathology assessment (Figure 3 and Figure 4). Copious irrigation of the surgical field was performed, and hemostasis was ensured.

**Figure 5:**
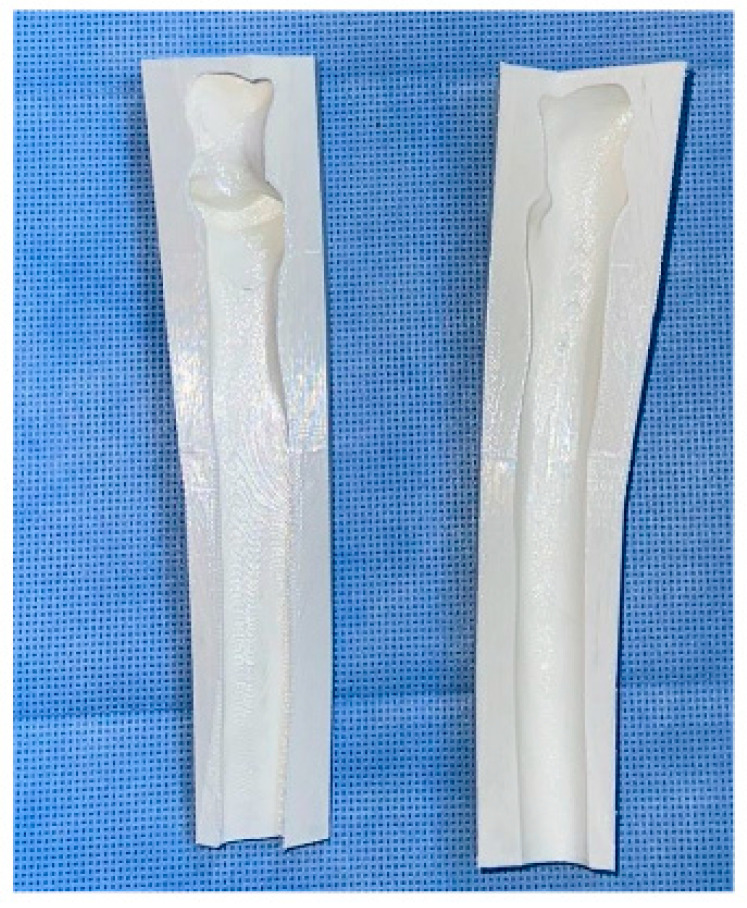
Three-dimensional (3D)-printed, custom-made ulnar cement mold.

Our attention was then focused on the reconstructive stage of the procedure, which was initially carried out by preparing the sterilized 3D-printed proximal ulnar cement mold (Figure 5). The mold was customized based on a mirrored CT scan of the contralateral ulna. The technical steps, including segmentation, mirror transformation, and mold design, were performed by a 3D printing expert to ensure anatomical accuracy. The mold was printed using an Anycubic Photon Mono X printer (Elegoo Mars 3, Elegoo Technology Co., Ltd., Shenzhen, China) and CHITUBOX software (CHITUBOX Basic V1.9.5 and CHITUBOX Pro V2.0), utilizing a non-medical, water-washable resin solely to fabricate the mold. This mold did not come into direct contact with the patient and was sterilized preoperatively using plasma sterilization. Intraoperatively, antibiotic-impregnated bone cement was prepared on the back table and cast into the mold to create a customized 3D-printed cement spacer replicating the patient’s proximal ulna. Once the cement hardened, the construct was drilled and fixed in proper alignment to the distal ulnar diaphysis using a 12-hole dynamic compression plate. Finally, we ensured adequate articulation of the proximal ulnar cement spacer with the ulnotrochlear and proximal radioulnar joints to maintain functional joint mechanics.

**Figure 6:**
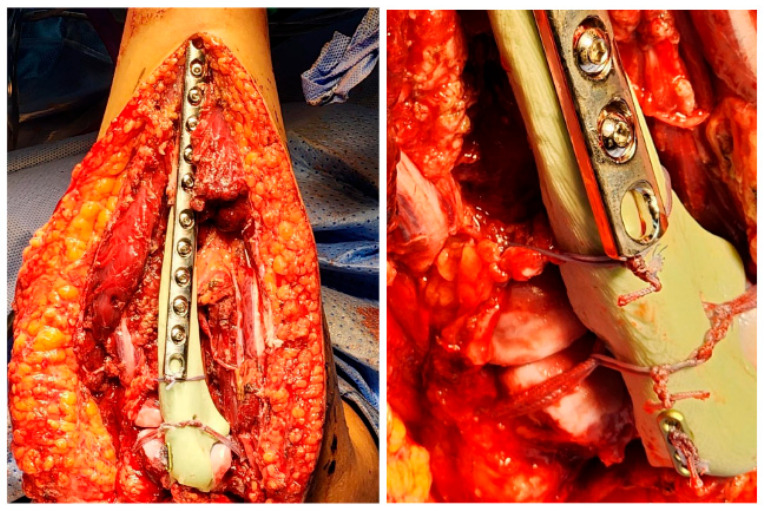
Ulna cement spacer fixation and reconstruction.

Our attention was subsequently shifted to the artificial ligamentous reconstruction of the elbow. The LUCL and MCL were reconstructed using FiberTape through tunnels made in the cement spacer. The remnants of the annular ligament were repaired using FiberWire. The triceps tendon was reattached to the spacer using an endobutton (Figure 6). Finally, the common flexor tendon was reattached to the humerus, followed by copious irrigation. Hemostasis was then achieved, and the wound was closed.

### 2.2. Postoperative Course and Histopathology

**Figure 7:**
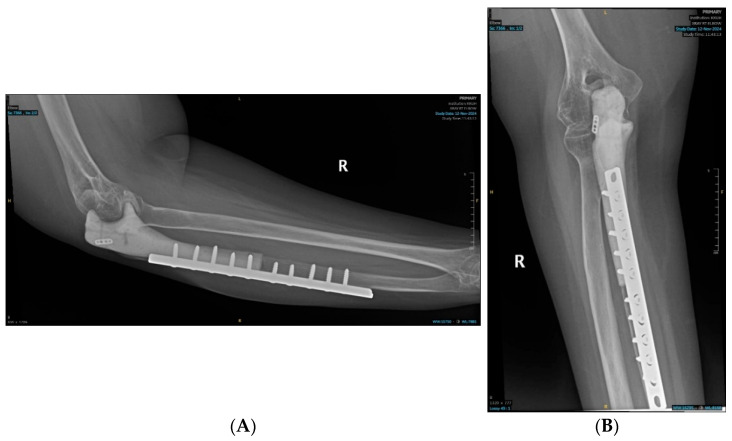
Postoperative radiographs of the ulnar reconstruction: (**A**) Ulnar implant—lateral view; (**B**) Ulnar Implant—Anteroposterior view.

Histopathology of the resected bone tumor revealed multiple osteoclastic giant cells, flanked by spindle-shaped mononuclear cells embedded in a fibrovascular stroma, and a paucity of osteoid, confirming the diagnosis of a high-grade GCRO of the right proximal ulna. The margins of resection did not show the presence of any tumor cells. Postoperative radiographs are shown in Figure 7.

The early postoperative course was uneventful, and the patient was discharged home after one week. The surgical wound healed primarily without surgical site infection or wound complications. Adjuvant chemotherapy was resumed three weeks postoperatively. Physiotherapy was planned and initiated shortly after surgery to restore the elbow functional range of motion (ROM) and elbow functionality. The patient was placed on a hinge elbow brace with gradual progression of elbow ROM. At six months follow-up, she had achieved an active ROM of 30 degrees of extension to 100 degrees of flexion, and 70 degrees of supination to 30 degrees of pronation. The patient continued to be followed regularly, and, at two years postoperatively, she maintained these functional outcomes with no reported complications such as fracture or implant failure.

## 3. Discussion

Giant cell-rich osteosarcoma (GCRO) is a rare subtype of osteosarcoma, accounting for only about 3% of all osteosarcomas [5]. This case is particularly notable due to the unusual site of involvement, with the tumor affecting the proximal ulna, a location that is very rare for primary bone sarcomas. Ulnar osteosarcomas account for less than 1% of all osteosarcomas and less than 2% of all osseous lesions [6]. This emphasizes the importance of considering such rare presentations in the differential diagnosis of suspected bone tumors of the elbow region. For patients with localized, non-metastatic osteosarcoma, systemic neoadjuvant and adjuvant chemotherapy, combined with local control via wide surgical resection and limb salvage reconstruction, remains the cornerstone of treatment [7]. In our case, the patient received neoadjuvant chemotherapy, followed by a wide surgical resection of the right proximal ulna with negative bone and soft tissue margins. Adjuvant chemotherapy was then administered as per standard treatment protocol after being discussed in a multidisciplinary tumor board meeting.

The reconstruction of a critical-size osteoarticular defect of the proximal ulna following tumor resection is challenging due to the complexity of anatomical structures and the limited available reconstructive options. One of the standout aspects of this case is the novel utilization of 3D printing technology for reconstructing the ulnar osteoarticular defect following tumor resection. Various methods for osteosarcoma reconstruction have been described, such as fibular autografts, allogenic bone grafts, arthrodesis, radius transposition, recycling of tumor bone, mega prosthesis, and free fibula transfer; nonetheless, these techniques can be technically challenging due to the complexity of precise anatomical alignment and ligamentous reconstruction [8,9,10,11,12]. Here, we utilized a 3D-printed cement spacer custom-molded from an inverted mirror of the patient’s own contralateral side. This innovative approach allowed for a highly precise and cost-effective solution for such complex reconstructive needs of the proximal ulna. Although some might argue that fibular grafts or off-the-shelf proximal ulnar endoprosthesis would have been a better option, our approach was driven by both the precision that 3D printing offers and its lower cost compared to more traditional methods. As healthcare costs continue to rise globally, it is crucial to explore affordable yet effective options for complex surgeries, and 3D printing offers an excellent solution. Moreover, the process is highly customizable, and we can tailor the prosthesis to the individual patient’s specific needs, ensuring the best possible functional outcome.

The applications of 3D printing in orthopedic surgery are becoming increasingly common for tumor resections, especially in cases like ours where the tumor affects a complex anatomical region. 3D-printed guides or prostheses can be specifically designed to match the patient’s unique anatomy, improving both the outcome of the surgical procedure and the precise fit of the reconstruction. These guides have been proven to be valuable in limb-salvage procedures, where conventional prosthetics may fail to provide an optimal fit. Previous reports on 3D-printed prostheses have focused on their use in radius, humerus, and hip reconstructions, but to our knowledge, this is the first case of using a 3D-printed cement mold and plate osteosynthesis composite for proximal ulnar reconstruction following GCRO resection [13,14,15]. Despite the perception of high cost associated with 3D-printing endoprosthetic reconstruction, the success of the relatively low-cost approach in our patient suggests that 3D printing utilization may offer a reliable and affordable alternative to more conventional, resource-intensive reconstructive options.

In this case, the patient’s postoperative recovery was uneventful, and by Day 8, she showed signs of healing with a range of motion (ROM) of 80° to 100° in the elbow. This outcome is consistent with previous reports, where patients who underwent elbow joint reconstruction following tumor excision achieved good functional outcomes. For instance, Guntur et al. reported similar results following distal humerus GCRO resections with elbow reconstruction [16], and Hao-Min Cui et al. noted positive results after elbow salvage surgeries involving autografts and prosthetic reconstructions [17]. Moreover, Apostolopoulos et al. demonstrated that using a total elbow prosthesis led to excellent results with full extension and good flexion in patients following proximal ulna resection [18]. Our patient also reported an improved ROM during further follow-up, with flexion–extension reaching from 30° to 100° after surgery, which is comparable to the functional outcomes seen in similar cases. A range of surgical options exists for reconstruction following proximal ulna resection, though each has specific limitations. Available reconstructive strategies include resection arthroplasty with or without reconstruction. Reconstructive approaches include the following: (1) use of a 3D-printed cement-molded articulating spacer with plate osteosynthesis (as used in our case); (2) radius neck-to-humerus trochlea transposition; (3) osteoarticular allograft reconstruction (not available in our region); and (4) tumor endoprosthesis-based elbow arthroplasty (also unavailable in our setting). The choice among these depends on various factors, including availability of implants, patient-specific anatomy, and urgency of systemic therapy initiation. Our decision to use a 3D-printed cement spacer was influenced by limited access to endoprosthetic or allograft solutions and the need to avoid delaying chemotherapy. This technique offered a cost-effective and rapidly deployable alternative. The subcutaneous nature of the proximal ulna presents a challenge, as infection risk is heightened with bulkier metallic implants. The patient has been followed for two years postoperatively with no complications and acceptable functional outcomes. While our technique may be considered either a definitive or temporary solution, revision to a more durable prosthetic reconstruction remains a future option if clinically indicated after completion of oncologic therapy.

## 4. Conclusions

In conclusion, the use of 3D printing for the reconstruction of the proximal ulna after GCRO resection offers a promising, cost-effective alternative to traditional methods. The ability to customize the implant to the patient’s specific anatomical needs ensures better surgical precision and functional outcomes. Given the positive results in this case, we believe that 3D printing should be considered as a viable option for complex reconstructions in osteosarcoma and other bone tumor surgeries. Further studies and clinical trials are needed to fully explore its potential; however, this case highlights its significant promise in orthopedic oncology.

## Data Availability

No datasets were generated or analyzed during the current study.
